# Exercise adherence and subjective well-being: the chain mediating roles of self-efficacy and outcome expectations

**DOI:** 10.3389/fpsyg.2026.1821014

**Published:** 2026-09-16

**Authors:** Jiaqi Liang, Jia Zhao

**Affiliations:** School of Physical Education, Shandong Normal University, Jinan, China

**Keywords:** exercise adherence, outcome expectations, self-efficacy, subjective wellbeing, university students

## Abstract

**Purpose:**

Although the rising prevalence of mental health issues among Chinese university students is influenced by multiple factors, it has been significantly associated with a lack of regular physical activity. The present study aims to examine the relationship between exercise adherence and subjective wellbeing among university students, specifically testing the mediating effects of self-efficacy and outcome expectations within a chain mediation framework.

**Methods:**

A questionnaire-based survey was conducted among a sample of 435 undergraduate students at Shandong Normal University (50.57% male, 49.43% female). The study employed validated instruments, including the Exercise Adherence Scale, the Self-Efficacy Scale, the Outcome Expectations Scale, and the Subjective Wellbeing Scale. Correlation and regression analyses, integrated with chain mediation modeling, were employed to examine the associations between exercise adherence, self-efficacy, outcome expectations, and subjective wellbeing.

**Results:**

Exercise adherence was significantly associated with subjective wellbeing both directly (effect value = 0.043) and indirectly through self-efficacy (effect value = 0.036), outcome expectations (effect value = 0.023), and the chain mediation of self-efficacy and outcome expectations (effect value = 0.012). Regression analysis revealed that exercise adherence significantly and positively predicted subjective wellbeing (β = 0.397, *p* < 0.001). After accounting for the indirect associations via self-efficacy and outcome expectations, exercise adherence remained significantly and positively associated with subjective wellbeing (β = 0.150, *p* = 0.002).

**Conclusion:**

Exercise adherence among Chinese university students is significantly and positively correlated with subjective wellbeing. This relationship is further linked to the chain mediating roles of self-efficacy and outcome expectations. Specifically, higher levels of exercise adherence are associated with greater self-efficacy, which relates to more positive outcome expectations, and both factors subsequently correlate with higher subjective wellbeing. Consequently, interventions focusing on these psychological variables may be beneficial in supporting the overall physical and mental health of university students.

## 1 Introduction

Subjective wellbeing is defined as individuals’ cognitive evaluations of their own lives and the affective responses associated with these evaluations. It is regarded as an important indicator of positive psychological development among college students (Diener et al., 1998; Zhang et al., 2024). In the context of contemporary social development, mental disorders have emerged as a leading contributor to the global burden of disease among young people. Most mental disorders first manifest during adolescence and early adulthood, typically between the ages of 12 and 24. Notably, approximately three-quarters of all lifetime mental disorders are diagnosed before the age of 25 (Nwachukwu et al., 2021; Tabor et al., 2021). Numerous studies have indicated that depressive and anxiety symptoms are the most prevalent mental health problems among college students (Liu et al., 2019). Consequently, mental health issues among college students warrant considerable attention. Depressive and anxiety symptoms in this population may be attributed to multiple factors, including intense academic demands, insufficient social support, and financial stress. These symptoms often manifest as communication difficulties and sleep disturbances, both of which have been shown to exert a substantial negative impact on individuals’ overall wellbeing (Liu et al., 2022; Zhou and Zhou, 2022). Wellbeing is widely regarded as a fundamental component of mental health. Within the field of wellbeing research, particular emphasis has been placed on subjective wellbeing, which is typically assessed using three core indicators: positive affect, negative affect, and life satisfaction (Yan and Zheng, 2006; Diener, 2013; Yu, 2022). College students’ exercise adherence, self-efficacy, and outcome expectations may play an important role in influencing this situation.

Physical exercise is widely recognized as an effective strategy for health promotion and offers advantages such as low cost and ease of implementation in alleviating psychological problems among students. Numerous studies have demonstrated that regular engagement in physical activity exerts a positive influence on individuals’ subjective wellbeing (Wang et al., 2023; An et al., 2024; Shi, 2025). A recent cross-sectional study by Xu et al. (2025) demonstrated that engaging in more than 2.5 h per week of moderate-intensity physical activity or more than 1.25 h per week of vigorous-intensity physical activity was associated with significantly higher levels of subjective wellbeing. Furthermore, empirical evidence suggests that physical activity significantly influences the social adaptation of university students (Liu Y. et al., 2023). For instance, Wang P. et al. (2025) demonstrated a direct positive correlation between physical exercise and children’s socio-emotional abilities, noting a stronger effect in males than in females. Similarly, Chaabene et al. (2025) reported that a multi-week intervention of low-to-moderate intensity aerobic exercise effectively reduces depressive symptoms and perceived stress among university students. Research also indicates that yoga practice is associated with immediate physiological changes, showing positive connections to cardiovascular function and emotional processing. This subsequently enhances social performance and overall quality of life (Herbert, 2022). Moreover, gender has emerged as a key moderating variable in health and fitness research (Bidmon and Terlutter, 2015). Previous studies show that men generally exhibit higher levels of physical activity, which correlates with increased subjective wellbeing and life satisfaction (Durau et al., 2022). Building on this, Liu Y. et al. (2023) emphasized that exercise promotes both physical health and cognitive function, thereby improving academic performance. Notably, their findings suggest a gender-specific time threshold for the best academic results: 2 h per day for boys and 1 h for girls (Liu G. et al., 2023). Exercise adherence is commonly defined as the extent to which individuals consistently engage in physical activity or exercise over a sustained period of time (Carron, 1982). Exercise has been shown to confer a wide range of beneficial effects. When performed consistently, physical exercise exerts a positive influence on mental health, with empirical evidence indicating improvements in executive function and cognitive performance (Wang H. et al., 2022). From a biological perspective, physical exercise is frequently linked to increases in gray matter volume within the prefrontal cortex and the hippocampus (Erickson et al., 2013). Such processes are closely tied to enhanced neural plasticity, improve cognitive function, memory, and emotional regulation, and thereby promote higher levels of mental health (Kandola et al., 2019; Frodl et al., 2020). The realization of these benefits is contingent upon long-term adherence to exercise regimens, underscoring the importance of maintaining regular physical activity. College students are at a critical stage of mental health development, making sustained engagement in physical exercise particularly important for this population. According to the National Student Physical Fitness and Health Report (2020), only 23.8% of Chinese college students meet the prescribed national fitness standards. This finding indicates a substantial lack of adherence to recommended physical activity guidelines among college students (Ministry of Education of the People’s Republic of China, 2020). This phenomenon not only reflects a lack of individual motivation but may also be influenced by environmental factors. According to the Social-Ecological Model, the diverse facilities and exercise opportunities provided by schools and communities serve as critical external factors that shape exercise behavior. Meanwhile, the family plays an essential role; support from the home environment and parental role modeling often convert external influence into the intrinsic motivation needed for physical activity (Zhang et al., 2022). It is therefore essential to examine the psychological mechanisms underlying exercise adherence among college students and to identify effective approaches for enhancing adherence. Such efforts are important not only for improving subjective wellbeing, but also for promoting both mental and physical health in this population.

Numerous studies have identified self-efficacy as a key factor in motivating college students to engage in physical exercise. In addition to these motivational effects, the psychological benefits derived from exercise behavior are also important, as they contribute to sustained participation in physical activity (Tang, 2019). Self-efficacy is defined as individuals’ subjective evaluations of, or beliefs in, their capability to perform specific behaviors in specific situations (Bandura, 1997). Yu et al. (2024) found that exercise motivation significantly and positively influences self-efficacy among undergraduate students. According to conservation of resources theory, self-efficacy plays a pivotal role in individuals’ management and regulation of emotional difficulties (Pei et al., 2024). The present study hypothesizes that self-efficacy plays a positive role in enhancing adaptability and reducing negative emotions, such as anxiety. It is further hypothesized that these effects contribute to improvements in individuals’ positive experiences and positive affect (Li et al., 2022; Wu et al., 2025). Individuals with higher levels of self-efficacy tend to exhibit greater confidence in daily life and report higher levels of subjective wellbeing. These findings suggest that self-efficacy may exert a significant positive influence on subjective wellbeing (Wang K. et al., 2022). Therefore, self-efficacy may moderate the relationship between exercise adherence and subjective wellbeing. According to social cognitive theory, Bandura emphasized the central roles of self-efficacy and outcome expectations in motivating individuals to regulate and control their behavior through belief perceptions and expectancy judgments, thereby influencing their behavioral choices (He and Guo, 2016; Huang, 2025). Outcome expectations refer to individuals’ judgments regarding the anticipated outcomes of their own behavior (Vroom, 1964). Bohlen et al. (2022) note that outcome expectations serve as effective predictors of exercise behavior; specifically, positive expectations regarding health benefits can drive higher levels of physical activity. In addition, Ding and Chen (2017) found that self-efficacy was positively correlated with outcome expectations, and that higher outcome expectations exerted a positive motivational effect on individuals. According to expectancy theory, individuals are more likely to engage in proactive behavior when they anticipate positive feedback and when their outcome expectations are met (Hmieleski et al., 2013). Therefore, consistently achieving attainable exercise goals and providing timely feedback are important for shaping and managing exercise-related outcome expectations.

Despite increasing attention to the link between physical activity and subjective wellbeing, the specific psychological mechanisms that transform long-term exercise adherence into improved wellbeing among college students remain underexplored. Previous research has largely examined mediating variables separately or focused on different intervention methods. Existing research still lacks sufficient insight into the combined and chain pathways of internal psychological drivers.

To address this gap, the present study incorporates self-efficacy and outcome expectations to clarify the complex mechanisms underlying this relationship. Based on the existing literature, the following hypotheses are proposed:

**Hypothesis 1**: Exercise adherence is positively associated with subjective wellbeing.

**Hypothesis 2**: Self-efficacy mediates the relationship between exercise adherence and subjective wellbeing.

**Hypothesis 3**: Outcome expectations mediate the relationship between exercise adherence and subjective wellbeing.

**Hypothesis 4**: Self-efficacy and outcome expectations play a chain mediating role in the association between exercise adherence and subjective wellbeing (Figure 1).

**FIGURE 1:**
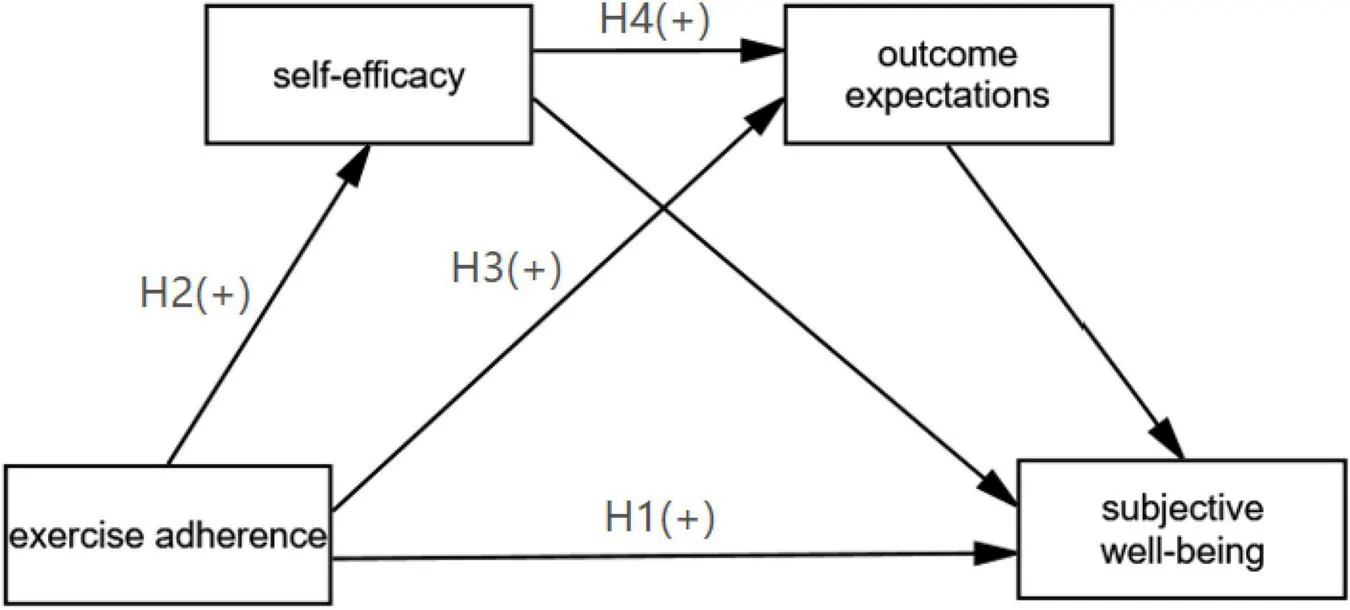
Hypothesized model of exercise adherence, self-efficacy, outcome expectations and subjective wellbeing.

## 2 Participants and methods

### 2.1 Participants

This study employed a cross-sectional survey design. The research protocol was approved by the Ethics Committee of the School of Physical Education, Shandong Normal University (Protocol Number: SDNUTYDW2025018; Date of Approval: 21 November 2025), and informed consent was obtained from all participants prior to data collection. Participants were recruited using a convenience sampling strategy through public elective courses and class-based WeChat groups to ensure broad departmental coverage. To enhance the representativeness of the sample, a quota sampling method was applied, utilizing demographic enrollment data (gender and grade level from freshman to senior) from the Academic Affairs Office of Shandong Normal University as a baseline. The survey was administered online via the Wenjuanxing platform^1^, taking approximately 15 min to complete. Furthermore, to maintain data quality and minimize potential bias, research assistants supervised the in-class data collection process in person.

A total of 486 students were invited to participate in the survey, and 441 responses were collected. To ensure data quality, data cleaning was performed to exclude invalid responses based on the following criteria: (1) uniform response patterns, (2) failure on reverse-coded attention checks, and (3) completion times of less than 3 min. Ultimately, 435 valid responses were included in the analysis, representing a 90.74% response rate and a 98.64% questionnaire validity rate (see Figure 2 for details).

**FIGURE 2:**
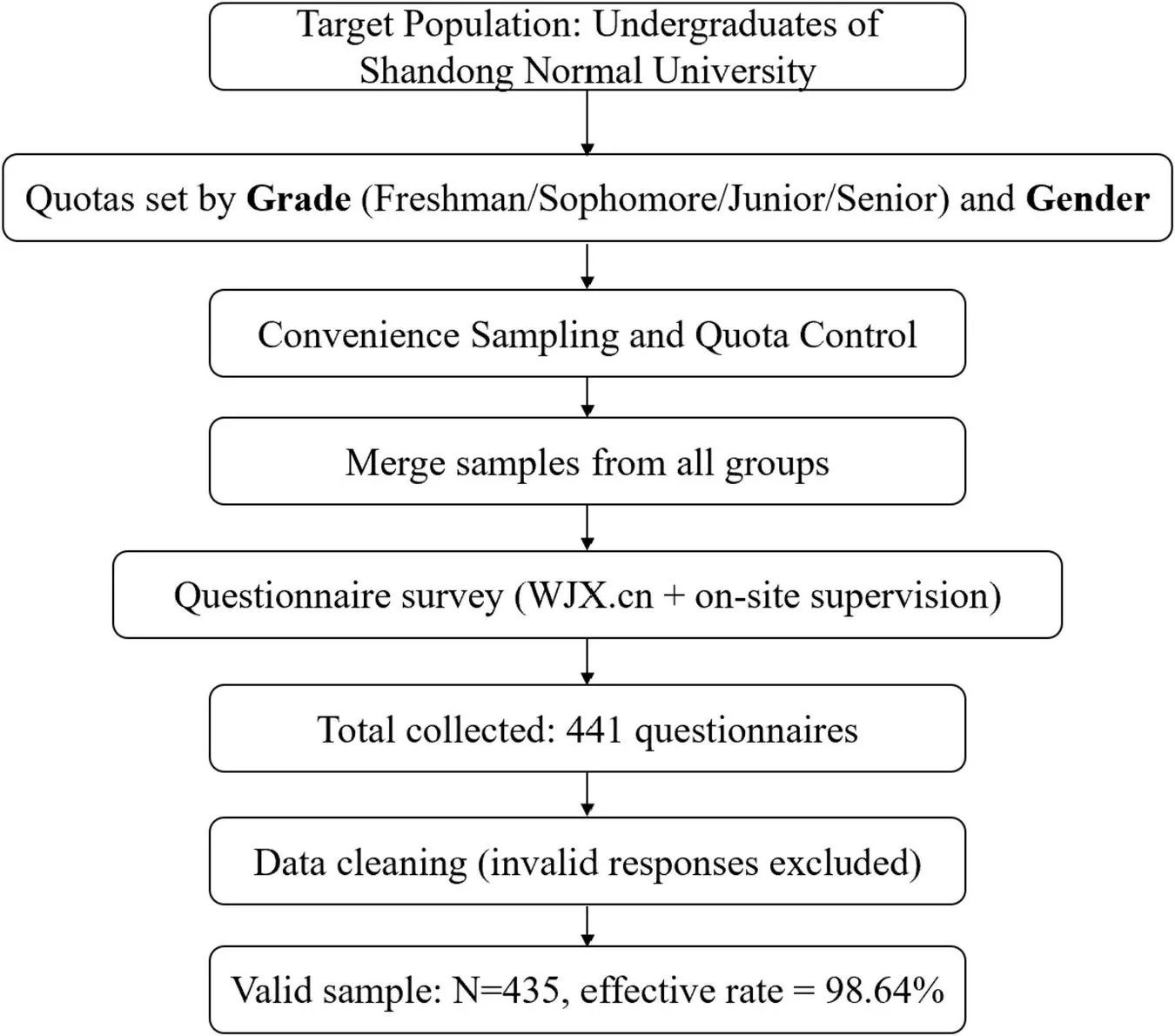
Flowchart of sample sampling procedure.

The demographic characteristics of the final sample are summarized as follows. Among the participants, 220 were male (50.57%) and 215 were female (49.43%). In terms of residential background, 203 students were from urban areas (46.67%) and 232 were from rural areas (53.33%). The sample comprised 146 freshmen (33.56%), 159 sophomores (36.55%), 121 juniors (27.82%), and nine seniors (2.07%). The first page of the questionnaire included an informed consent statement to ensure voluntary participation, along with an information sheet describing the purpose of the study and emphasizing the anonymity and confidentiality of responses, as well as the authenticity of participants’ answers.

### 2.2 Measuring tools

#### 2.2.1 Exercise adherence scale

Exercise adherence was assessed using the Exercise Adherence Scale developed by Wang et al. (2016). The scale comprises three dimensions: behavioral habits, effort investment, and emotional experience, and includes a total of 14 items. All items are rated on a five-point Likert scale ranging from 1 (completely inconsistent) to 5 (completely consistent). A representative sample item is “I exercise for at least an hour every time.” Confirmatory factor analysis indicated an acceptable model fit (CMIN/DF = 2.504, RMSEA = 0.059, CFI = 0.982, TLI = 0.977, NFI = 0.970), demonstrating good structural validity. The scale exhibited excellent internal consistency, with a Cronbach’s α coefficient of 0.958.

#### 2.2.2 Self-Efficacy Scale

Self-efficacy was assessed using the General Self-Efficacy Scale originally developed by Schwarzer et al. and subsequently translated and revised by Wang et al. (2001). The present study utilizes the General Self-Efficacy Scale (GSES) for two primary reasons. First, the study focuses on subjective wellbeing, which represents a comprehensive evaluation of life satisfaction. The GSES measures an individual’s general confidence in overcoming various challenges, making the two constructs highly consistent in nature. Second, the study examines not only the physical act of exercise but also its “transfer effect”—the ability of exercise to enhance students’ confidence in dealing with daily life challenges. Therefore, the GSES is a more effective tool for observing the psychological transition from physical activity to overall psychological wellbeing.

The scale is designed to assess individuals’ perceived ability or confidence in performing specific behaviors. It comprises 10 items rated on a four-point Likert scale ranging from 1 (*completely incorrect*) to 4 (*completely correct*), with higher scores indicating greater self-efficacy. For example, a sample item is “If I do my best, I can always sort things out.” The scale demonstrated excellent internal consistency, with a Cronbach’s α coefficient of 0.977. Confirmatory factor analysis indicated an acceptable model fit (CMIN/DF = 2.613, CFI = 0.995, TLI = 0.988, RMSEA = 0.061, NFI = 0.991), supporting good structural validity.

#### 2.2.3 Outcome expectation scale

Outcome expectations were assessed using the 15-item Multidimensional Outcome Expectations for Exercise Scale (MOEES), developed by Wojcicki et al. (2009). The scale is used to evaluate students’ subjective perceptions of the likelihood of obtaining specific benefits from regular physical activity, as well as the extent to which they endorse corresponding exercise-related outcome expectations (Wojcicki et al., 2009). The scale comprises three dimensions: physical, social, and self-evaluative outcomes. Responses are recorded using a five-point Likert scale ranging from 1 (*completely disagree*) to 5 (*completely agree*), yielding total scores between 15 and 75. A representative sample item is “Exercise helps me manage my weight better.” In the present study, the MOEES demonstrated good internal consistency (Cronbach’s α = 0.96), which is consistent with previous findings (α = 0.96) (Farren et al., 2017). Confirmatory factor analysis indicated an acceptable model fit (CMIN/DF = 3.734, CFI = 0.970, TLI = 0.957, RMSEA = 0.079, NFI = 0.960), supporting adequate structural validity.

#### 2.2.4 Subjective wellbeing scale

Subjective wellbeing was assessed using the Well-Being Index developed by Campbell et al. (1976). The scale is a self-report instrument comprising two dimensions: an overall affect index and a life satisfaction index. The overall affect index consists of eight items, whereas the life satisfaction index includes a single item. All items are rated on a seven-point Likert scale. The wellbeing index is calculated by summing the mean score of the overall affect index (weighted at 1.0) and the mean score of the life satisfaction index (weighted at 1.1). Higher total scores indicate greater subjective wellbeing. A sample item from the overall affect index asks respondents to rate how they feel on a spectrum, such as “Boring” to “Interesting.” Previous research has demonstrated the satisfactory applicability of this questionnaire among Chinese university students (Ye and Guo, 2023). In the present study, the scale demonstrated excellent internal consistency, with a Cronbach’s α coefficient of 0.972. Confirmatory factor analysis indicated an acceptable model fit (CMIN/DF = 2.81, CFI = 0.992, TLI = 0.987, RMSEA = 0.065, NFI = 0.988), supporting good structural validity.

### 2.3 Data analysis

Descriptive statistics and correlation analyses were performed using SPSS version 27.0. To test the chain mediation model, the PROCESS macro (Model 6) was employed to examine the associations and indirect pathways between variables (Preacher and Hayes, 2008). In the proposed model, exercise adherence was designated as the independent variable, subjective wellbeing as the dependent variable, and self-efficacy and outcome expectations as the chain mediators. The 95% confidence interval (CI) was estimated based on 5,000 bootstrap samples. A chain mediation effect was considered statistically significant if the 95% CI did not include zero (Xiao et al., 2022).

*Post hoc* power analysis was conducted using G*Power 3.1 software to evaluate the statistical reliability of the sample size. For the multiple regression model (with subjective wellbeing as the dependent variable), the R^2^ value of 0.304 corresponds to an effect size of f^2^ = 0.437. With a significance level of α = 0.05, six predictor variables, and a sample size of *N* = 435, the statistical power exceeded 0.99. This indicates that the current sample size provides sufficient statistical power and reliability for the study.

## 3 Results

### 3.1 Common method bias test

Procedural controls were initially implemented to mitigate common method bias. These included the incorporation of reverse-scored items within the scales and the randomization of scale order. Additionally, participants were informed about the anonymity of their responses prior to questionnaire administration. Subsequently, Harman’s single-factor test was conducted to assess the presence of common method bias. The analysis revealed seven factors with eigenvalues greater than 1, with the first factor accounting for 44.12% of the total variance—below the critical threshold of 50%. These results indicate that common method bias was not a significant concern in the data (Fuller et al., 2016).

### 3.2 Correlation analysis

The present study employed SPSS Statistics 27.0 to perform correlation analyses, with the results for each variable summarized in Table 1. The findings revealed significant positive correlations between exercise adherence and self-efficacy, outcome expectations, and subjective wellbeing (*r* = 0.475, *r* = 0.526, and *r* = 0.403, respectively). Additionally, self-efficacy was significantly and positively correlated with both outcome expectations and subjective wellbeing (*r* = 0.551 and *r* = 0.474, respectively). Lastly, outcome expectations showed a significant positive correlation with subjective wellbeing (*r* = 0.468). Additionally, gender, grade, and place of residence (urban/rural) were included as covariates to control for their potential influence on the results.

**TABLE 1:** Statistics of Pearson correlation coefficient.

Variable	M ± SD	1	2	3	4	5	6	7
1. Gender	1.49 ± 0.50	1	–	–	–	–	–	–
2. Grade	1.98 ± 0.84	−0.411**	1	–	–	–	–	–
3. Place of residence	1.53 ± 0.50	0.012*	−0.018	1	–	–	–	–
4. Exercise adherence	59.12 ± 10.20	0.035	−0.027	−0.056	1	–	–	–
5. Self-efficacy	27.33 ± 8.81	0.071	−0.125**	−0.071	0.475**	1	–	–
6. Outcome expectations	63.43 ± 9.44	0.008	−0.034	−0.144**	0.526**	0.551**	1	–
7. Subjective wellbeing	11.51 ± 2.91	0.005	−0.071	−0.100*	0.403**	0.474**	0.468**	1

*P < 0.05,

**p < 0.01.

### 3.3 Regression analysis of exercise adherence, self-efficacy, outcome expectations, and subjective wellbeing

This study investigated the chain mediating roles of self-efficacy and outcome expectations in the association between exercise adherence and subjective wellbeing among college students. As shown in Table 2, exercise adherence was significantly and positively associated with subjective wellbeing (β = 0.397, *p* < 0.001). This association remained significant, though attenuated, after including self-efficacy and outcome expectations as chain mediators (β = 0.150, *p* = 0.002). Additionally, exercise adherence demonstrated significant positive associations with self-efficacy and outcome expectations, with standardized coefficients of 0.469 (*p* < 0.001) and 0.337 (*p* < 0.001), respectively. The analysis also revealed a significant positive association between self-efficacy and outcome expectations, with a path coefficient of 0.387 (*p* < 0.001).

**TABLE 2:** Regression analysis of the multiple mediation model.

Dependent variable	Independent variable	*R*	R^2^	*F*	β	SE	*t*	*P*	95% CI [LL, UL]
SW	Gender	0.416	0.173	22.517	−0.040	0.279	−0.833	0.405	[−0.782, 0.316]
	Grade				−0.078	0.167	−1.621	0.106	[−0.600, 0.058]
	Place of residence				−0.079	0.256	−1.793	0.074	[−0.961, 0.044]
	EA				0.397	0.013	9.042	<0.001	[0.089, 0.138]
SW	Gender	0.551	0.304	31.141	−0.038	0.257	−0.851	0.395	[−0.724, 0.287]
	Grade				−0.042	0.155	−0.933	0.351	[−0.449, 0.160]
	Place of residence				−0.039	0.237	−0.960	0.338	[−0.694, 0.239]
	EA				0.150	0.014	3.050	0.002	[0.015, 0.070]
	SE				0.268	0.017	5.309	<0.001	[0.056, 0.121]
	OE				0.235	0.016	4.492	<0.001	[0.041, 0.106]
OE	Gender	0.636	0.404	58.193	−0.026	0.771	−0.629	0.530	[−2.000, 1.031]
	Grade				0.011	0.465	0.279	0.780	[−0.784, 1.044]
	Place of residence				−0.097	0.707	−2.602	0.010	[−3.228, −0.450]
	EA				0.337	0.039	7.959	<0.001	[0.235, 0.389]
	SE				0.387	0.046	9.055	<0.001	[0.325, 0.505]
SE	Gender	0.490	0.240	34.018	0.010	0.811	0.218	0.828	[−1.418, 1.772]
	Grade				−0.109	0.486	−2.366	0.018	[−2.105, −0.195]
	Place of residence				−0.047	0.742	−1.112	0.267	[−2.285, 0.634]
	EA				0.469	0.036	11.13	<0.001	[0.333, 0.476]

*N* = 435; β, standardized regression coefficient; SE, standard error; CI, confidence interval; LL, lower limit; UL, upper limit.

### 3.4 Test of the mediating effects of self-efficacy and outcome expectations between exercise adherence and subjective wellbeing

To test Hypothesis 3, a chain mediation analysis was performed using PROCESS Model 6 in SPSS. The results, summarized in Table 3, revealed a significant positive direct association between exercise adherence and subjective wellbeing (estimate = 0.043, 95% CI [0.0152, 0.0702]), accounting for 37.17% of the total association.

**TABLE 3:** Test results of bootstrap mediation effect.

Effect path	Effect value	Boot	Boot CI	Boot CI	Effect percentage
		Standard error	Lower limit	Upper limit	
Total effect	0.113	0.013	0.0886	0.1377	100%
Direct effect	0.043	0.014	0.0152	0.0702	37.17%
Total mediating effect	0.071	0.010	0.0519	0.0911	62.83%
EA-SE-SW (Ind1)	0.036	0.008	0.0209	0.0541	31.86%
EA-OE-SW (Ind2)	0.023	0.006	0.0112	0.0354	20.35%
EA-SE-OE-SW (Ind3)	0.012	0.004	0.0058	0.0200	10.62%

Effect value refers to the unstandardized coefficients (B).

Regarding the indirect pathways, the mediation linking exercise adherence to subjective wellbeing via the mediator self-efficacy (EA→SE→SW) was significant (estimate = 0.036, 95% CI [0.0209, 0.0541]), representing 31.86% of the total indirect association. Similarly, the mediation pathway via the mediator outcome expectations (EA→OE→SW) was significant (estimate = 0.023, 95% CI [0.0112, 0.0354]), accounting for 20.35% of the total indirect association. Finally, the chain mediation pathway (EA→SE→OE→SW) was also significant (estimate = 0.012, 95% CI [0.0058, 0.0200]), contributing 10.62% to the total indirect association.

## 4 Discussion

This study employed a cross-sectional design to investigate the mechanisms through which exercise adherence influences subjective wellbeing, with particular emphasis on the mediating roles of self-efficacy and outcome expectations. The following discussion will elaborate on the research hypotheses, interpret the findings in light of relevant theoretical frameworks, and highlight their practical and theoretical implications.

### 4.1 The direct association between exercise adherence and subjective wellbeing

The present findings reveal a robust positive correlation between exercise adherence and subjective wellbeing among college students, consistent with previous research in this domain (Tang, 2019; Toros et al., 2023; Ye and Guo, 2023). The positive impact of exercise adherence on subjective wellbeing can be attributed to several underlying mechanisms. Regular physical activity has been shown, at the biological level, to stimulate brain function, enhance cognitive performance, and regulate the secretion of neurotransmitters and hormones such as endorphins and dopamine. These physiological responses contribute significantly to the improvement of overall wellbeing (Qiao and Fan, 2020; Festa et al., 2023). In addition to physiological benefits, adherence to exercise regimens has been shown to enhance subjective wellbeing by fostering social support, which cultivates feelings of understanding and respect, thereby promoting emotional stability (Fredrickson, 2001). Existing research indicates that structured physical exercise has a more pronounced positive effect on subjective wellbeing compared to unstructured physical activity (Buecker et al., 2021). Furthermore, research has demonstrated that regular participation in physical exercise improves fitness levels, promotes overall physical health, and enhances subjective wellbeing (Wang et al., 2023). Consequently, sustained adherence to exercise regimens may positively impact the subjective wellbeing of college students.

### 4.2 The mediating role of self-efficacy

The findings of this study support Hypothesis 2, with mediation analysis revealing that self-efficacy serves as a significant mediator in the association between exercise adherence and subjective wellbeing. Specifically, higher levels of exercise adherence were found to be positively associated with stronger self-efficacy among college students, which in turn was related to enhanced subjective wellbeing. This pattern is consistent with the correlations observed in previous studies (Desharnais et al., 1986; Guo et al., 2024). Furthermore, Liu established that self-efficacy acts as a critical mediator linking physical exercise to mental health. Guo et al. (2024) found a significant positive correlation between physical activity and self-efficacy among college students (Liu, 2020). Kan and Xie (2024) found that exercise frequency is positively associated with both self-efficacy and life satisfaction. Various forms of exercise have different impacts on psychological and cognitive health. For instance, Faigenbaum et al. (2013) found that compared to aerobic exercise, resistance training is associated with reduced depressive symptoms and higher levels of self-efficacy, suggesting a stronger link to adolescent mental health. Similarly, Chaabene et al. (2025) reported that while the positive correlations between aerobic exercise and mental health are well-documented, resistance training may demonstrate more pronounced associations with mental health and cognitive function. While running, cycling, badminton, and basketball are the most prevalent physical activities among young people, higher exercise frequency is also significantly associated with greater life satisfaction (Ministry of Education of the People’s Republic of China, 2025). Life satisfaction is regarded as a key cognitive component of subjective wellbeing (Shin and Johnson, 1978). Consequently, higher levels of exercise adherence among college students correspond to greater self-efficacy and more positive emotions, which are ultimately linked to higher levels of subjective wellbeing.

### 4.3 The mediating role of outcome expectations

The findings of this study support Hypothesis 3, indicating that outcome expectations serve as a significant mediator in the association between exercise adherence and subjective wellbeing among college students. Specifically, higher exercise outcome expectations are associated with a stronger linkage between exercise adherence and subjective wellbeing. This result aligns with previous research in the field (Bohlen et al., 2022; Wang N. et al., 2025); however, this finding contrasts with the conclusions drawn by Malherbe, highlighting a divergence in the understanding of the role of outcome expectations (Malherbe et al., 2003). Kawaji et al. (2022) posited that outcome expectations represent a psychological factor that linked to physical activity. Outcome expectations are defined as an individual’s understanding of the benefits associated with physical activity, along with the anticipated consequences stemming from it. Regular exercise is associated with positive outcomes in physical appearance, mood, and athletic performance, and individuals maintain specific expectations regarding these benefits. Research by Cruz et al. (2013) indicates that individuals often perceive higher positive outcome expectations following exercise, with more frequent participation being linked to stronger expectations of derived benefits. Previous research indicates that higher levels of consistency in physical activity are positively associated with stronger expectations regarding exercise outcomes among college students (Bohlen et al., 2022). When individuals believe that a specific activity will yield positive expectations, they tend to develop a strong intention to participate. This behavioral intention, in conjunction with actual physical activity, serves as a significant predictor of subjective wellbeing, which is associated with enhanced mental health among adolescents (Batista et al., 2026). Klusmann et al. (2016) found that positive exercise experiences correspond to the fulfillment of emotional expectations, which relates to improved psychological experiences and higher subjective wellbeing. Consequently, exercise consistency shows a positive correlation with favorable experiences and higher expectations, both of which are linked to enhanced subjective wellbeing. Moreover, lower expectations of negative outcomes are associated with more persistent participation (Zhou et al., 2021). According to the Health Action Process Approach (HAPA), task self-efficacy, positive outcome expectations, and risk perception are essential prerequisites for forming action intentions (Perrier, 2012). Wang N. et al. (2025) demonstrated that positive expectations regarding physical activity lead to higher levels of exercise participation among college students.

Therefore, exercise adherence is positively associated with intrinsic motivation and higher outcome expectations, which in turn relates to a greater likelihood of achieving fitness goals and enhanced subjective wellbeing.

### 4.4 The chain mediating role of self-efficacy and outcome expectations between exercise adherence and subjective wellbeing

The fourth objective of this study was to examine the chain mediation effect of self-efficacy and outcome expectations in the association between exercise adherence and subjective wellbeing. According to Social Cognitive Theory, human behavior is viewed as the result of a dynamic interaction between individual and environmental factors. Furthermore, the theory posits that behavior directly influences an individual’s self-efficacy, outcome expectations, and self-regulatory processes (He and Guo, 2016). As hypothesized by Bandura, self-efficacy functions as a causal antecedent to outcome expectations (Bandura, 1986). However, from a motivational perspective, the initiation of a new behavior is more strongly associated with outcome expectations than with self-efficacy. For instance, when adhering to an exercise regimen, an individual’s motivation to achieve specific goals—such as improving physical fitness—often plays a more dominant role than the influence of self-efficacy. As the individual persists in regular physical activity, this goal-oriented motivation tends to be further reinforced and strengthened (Gao et al., 2008). The primary function of self-efficacy lies in behavior regulation, as reflected in the strength of an individual’s confidence in their capabilities (Bandura, 1997). Generally, people tend to develop greater confidence in their ability to achieve positive physical health outcomes through exercise, rather than focusing on preventing negative outcomes (Karvinen and Vallance, 2015). Higher self-efficacy is associated with increasing outcome expectations. When individuals possess greater confidence in their abilities, their expectations of attaining beneficial outcomes also rise; conversely, those with lower self-confidence tend to report diminished expectations regarding the benefits of physical exercise (Maddux et al., 1982). Boykin (1996) proposed that self-efficacy, outcome expectations, and exercise adherence are significantly interrelated. When individuals perceive that a specific activity is associated with positive outcomes, these expectations can significantly predict subjective wellbeing. Outcome expectations involve positive psychological effects. When these expectations are met, there is a positive link to both the maintenance of exercise behavior and the enhancement of subjective wellbeing, both of which relate to broader psychological health benefits (Klusmann et al., 2016). Additionally, adherence to regular exercise regimens is often accompanied by the release of endorphins—natural analgesics that are closely connected to improved mood and further related to enhanced subjective wellbeing (Qi et al., 2020).

In conclusion, exercise adherence among college students is significantly and positively associated with subjective wellbeing, primarily through the chain mediation of self-efficacy and outcome expectations. Sustained engagement in regular exercise relates to higher students’ self-efficacy, reflecting greater confidence and positive expectations regarding exercise outcomes, which collectively correspond to higher levels of subjective wellbeing. Furthermore, although the model developed in this study has been validated across the overall sample, significant gender-specific differences may exist in the associative pathways between variables. Recent literature suggests that, for men, participation in physical exercise is closely linked to higher levels of subjective wellbeing (Zhao et al., 2026). In contrast, research on female university students indicates that exercise can sometimes be associated with more pronounced psychological distress and fatigue. The correlation between regular physical exercise and mental health in women is often more complex, potentially intertwined with the interplay of multiple physical (e.g., body image) and psychological factors (e.g., self-efficacy and engagement in social activities) (Levante et al., 2024). Consequently, examining these nuanced gender variations and their underlying mechanisms should be a valuable direction for future research.

## 5 Limitations

The present study has several limitations. First, while it proposes that exercise adherence influences subjective wellbeing, the possibility of reverse causality—where individuals with higher subjective wellbeing are more likely to maintain exercise adherence—cannot be ruled out. Therefore, future research should employ longitudinal designs or rigorous experimental methods to more precisely clarify the directional relationship between exercise adherence and subjective wellbeing. Second, the sample was restricted to undergraduate students from Shandong Normal University, representing a single geographic region and institution type. Future studies should consider expanding the sample to include multiple regions and diverse university settings to enhance the generalizability of findings across broader populations. Third, subsequent research could incorporate additional variables such as social support, psychological resilience, and self-esteem to deepen understanding of the multifaceted determinants of subjective wellbeing (Levante et al., 2024). Finally, although this study established quantitative relationships among exercise adherence, self-efficacy, outcome expectations, and subjective wellbeing, it did not employ qualitative methods—such as in-depth interviews—to explore students’ psychological experiences in greater depth. Future investigations could benefit from qualitative approaches, including grounded theory or case studies, to yield richer, more nuanced insights into the mental health of college students.

## 6 Suggestions

Based on the correlations identified in this study, higher education institutions could consider the potential links between lifelong physical education and student development. Diversifying sports activities is associated with higher levels of exercise adherence, self-efficacy, and subjective wellbeing among students. Additionally, encouraging regular fitness routines is closely connected with the maintenance of sustainable exercise habits.

Although this study established a statistical association between exercise adherence and subjective wellbeing, it does not imply a causal relationship. Therefore, these findings should be interpreted with caution. Future research should aim to include more diverse samples to further explore the factors linked to college students’ exercise habits and mental health.

## 7 Conclusion

In summary, college students’ adherence to exercise regimens is positively associated with their subjective wellbeing, with this relationship shaped by both the independent mediating roles of self-efficacy and outcome expectations, as well as their sequential chain mediating effect. The findings confirm a significant positive correlation between exercise adherence and subjective wellbeing, revealing indirect pathways through which adherence links to wellbeing: via self-efficacy alone, via outcome expectations alone, and via the combined chain of self-efficacy followed by outcome expectations. Specifically, higher levels of exercise adherence among college students are associated with greater self-efficacy and more positive outcome expectations, which in turn correlate with higher subjective wellbeing and, ultimately, better physical and mental health. Collectively, these results provide valuable insights into the nuanced correlations between exercise adherence and subjective wellbeing among Chinese college students, shedding light on the interconnected psychological mechanisms that underpin the exercise-wellbeing link in this population.

## Data Availability

The raw data supporting the conclusions of this article will be made available by the authors, without undue reservation.

## Footnotes

1
https://www.wjx.cn/
